# The immune contexture of hepatocellular carcinoma predicts clinical outcome

**DOI:** 10.1038/s41598-018-21937-2

**Published:** 2018-03-29

**Authors:** Friedrich Foerster, Moritz Hess, Aslihan Gerhold-Ay, Jens Uwe Marquardt, Diana Becker, Peter Robert Galle, Detlef Schuppan, Harald Binder, Ernesto Bockamp

**Affiliations:** 1 Institute of Translational Immunology and Research Centre for Immunotherapy, University Medical Centre of the Johannes Gutenberg University Mainz, Mainz, Germany; 2 First Department of Medicine, University Medical Centre of the Johannes Gutenberg University Mainz, Mainz, Germany; 3 Institute of Medical Biostatistics, Epidemiology and Informatics (IMBEI), University Medical Centre of the Johannes Gutenberg University Mainz, Mainz, Germany; 4 Division of Gastroenterology, Beth Israel Deaconess Medical Center, Harvard Medical School, Boston, USA

## Abstract

The general relevance of the immune system for cancer development and therapy is increasingly recognized. However and although the immune contexture of most human cancer types has been determined, a global characterisation of the immune tumour microenvironment in hepatocellular carcinoma (HCC) is lacking. Equally, differences in the immune contexture of HCC between different patient subgroups and its effect on survival remain to be established. Here we report an *in silico* analysis of the immune contexture of human HCC. Using large deep sequencing HCC tumour, adjacent non-tumour and healthy liver high-dimensional data sets, we were able to reveal previously unrecognized differences in the immune contexture of HCC. Strikingly, we found that different etiologies and HCC stages were not associated with major changes in the immune contexture. In contrast, the presence of T cells and cytotoxic cells as well as the absence of macrophages and Th2 cells positively correlated with patient survival. Based on these novel findings, we developed a prognostic score that accurately distinguishes between patients with good and poor survival. Our study provides the first global characterisation of the immune contexture of HCC and will have direct implications for future HCC therapies.

## Introduction

Hepatocellular carcinoma (HCC) is one of the most frequent and lethal human cancers, and its incidence is rising^1,2^. HCC has an overall dismal prognosis and only when diagnosed early, surgery and ablative therapies may offer a cure^3^. In most cases, however, HCC is diagnosed at an advanced stage, when multi tyrosine kinase inhibitors (sorafenib^4^ or regorafenib^5^) and most recently the immune checkpoint inhibitor nivolumab^6,7^ or best supportive care remain the only available treatment options. To develop more effective therapies and to identify factors that determine patient survival, a better mechanistic understanding of HCC is urgently needed.

The immune system strongly influences cancer development^8,9^. Compelling evidence indicates that tumours evade destruction by suppressing the host’s immune system^10^. It is now generally accepted that tumour-immune cell interactions are highly relevant for patient survival and that the immune contexture of tumours represents a therapeutic target for improving clinical outcome^9,11–14^. Since HCC typically arises in the background of chronic inflammation (caused by alcohol consumption, virus infections or non-alcoholic fatty liver disease)^15^, it will be crucial to understand the pro- and anti-tumour function of the immune system in a chronically inflamed but tumour-tolerant microenvironment. So far, studies on the immune tumour microenvironment (TME) of HCC have been limited to biochemical, flow cytometric or microscopic methodologies that are often cumbersome and only provide information about a reduced number of markers^16–19^. By contrast, next generation bioinformatics analysis of high-dimensional deep sequencing data offers the unique opportunity to comprehensively analyse transcriptional immune-regulatory networks and to accurately determine the different immune cell types that invade the TME. Using next generation bioinformatics, several papers reported the effect of the tumour immune contexture on patient survival for most human cancers^11–13^. Strikingly and although HCC is one of the major human cancer malignancies, a comprehensive immunome analysis for HCC is still missing. Equally, it remains unknown whether overall immune cell numbers or the infiltration of specific immune cell types predict patient survival in HCC. Moreover and central to stratifying future treatment regimes, it is completely unknown if human HCCs of different etiology and stage differ in their immune TME. To guide current immune therapy trials and to improve future treatment options, detailed insight into the relationship between immune cell infiltration and clinical outcome will be needed^20,21^.

Combining high-throughput RNA-sequencing (RNA-Seq) HCC patient data with next generation bioinformatics, we here report the first *in silico* immunome characterisation of human HCC. The results of this analysis clearly demonstrate that the nature and composition of tumour-infiltrating immune cells predict patient survival.

## Results

### Patient characteristics

We assembled individual transcriptomes from HCC and matched non-tumour samples using RNA-Seq data from The Cancer Genome Atlas (TCGA)^22^. After manually curating all data with respect to relevant clinical parameters (gender, ethnicity, etiology, tumour stage, performance, liver function and survival), 371 HCC tumour samples (HCC-T) and 50 matched non-tumour samples (HCC-NT) were included in the analysis. The majority of the selected patients were male (68% male/32% female) and Asian or Caucasian (45% and 49%, respectively). The most common disease etiology was hepatitis B (32%) followed by alcohol consumption (25%) and hepatitis C (11%). According to the classification of malignant tumours (TNM classification), half of the patients had HCC stage I (49%), one quarter stage II (25%) and one quarter stage III or IV (26%). 90% of patients were R0-resected (no cancer cells at the resection margin) and in 5%, residual tumour (R1 (microscopic positive margin); and R2 (macroscopic positive margin)) was found in the resection margins. Two thirds (70%) had unaffected regional lymph nodes and three quarters (73%) were free of distant metastasis, while lymph node or distant metastasis were detected in only 1% each. Two thirds (65%) were free of vascular invasion, one third (30%) had micro-vascular and only a minority (5%) macro-vascular invasion. 87% of patients had a good or very good Eastern Cooperative Oncology Group (ECOG) performance status (ECOG 0 and 1) and 91% unimpaired liver function (Child-Pugh-Score A). The clinical characteristics of all patients as provided by TCGA are listed in Supplementary Table 1.

### Although HCCs are surrounded by hyper-inflamed T helper cell 1 (Th1)-type non-tumour liver tissue, the tumour microenvironment is Th2-skewed and shows strongly reduced cytolytic and antigen presenting activity

**Figure 1:**
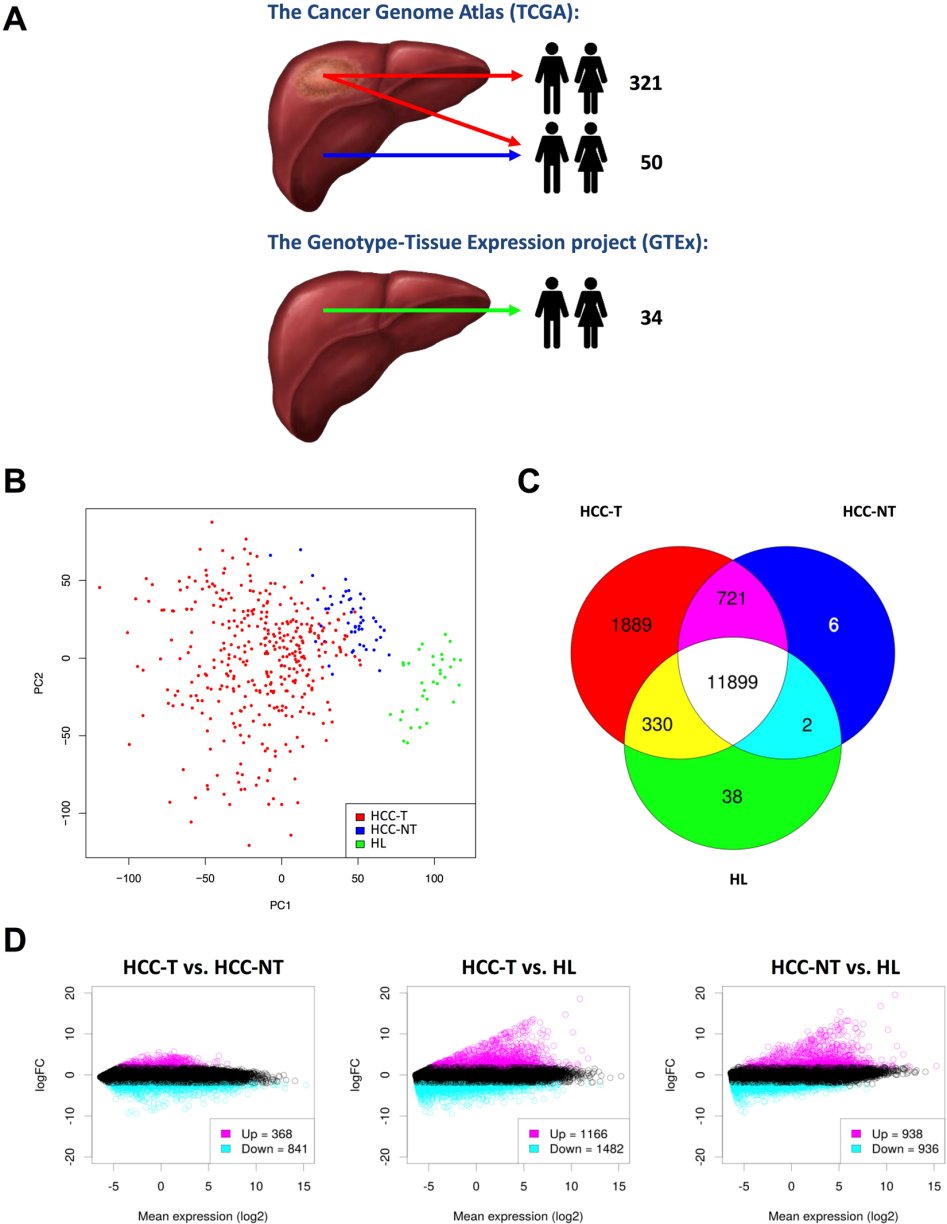
Transcriptomes from HCC and surrounding tissue are more closely related to each other than to healthy liver. (**A**) Schematic representation of data acquisition and analysis. Messenger RNA levels from HCC (371 patients), matched surrounding tissue (50 patients) and healthy livers (34 individuals) were obtained from The Cancer Genome Atlas (TCGA) and the Genotype-Tissue Expression project (GTEx). (**B**) Differentiation of gene expression between HCC (HCC-T), tissue adjacent to HCC (HCC-NT) and healthy liver samples (HL). Samples are plotted based on their scores for the two variance components that explained most of the overall gene expression variation. The variance components were determined by a principal component analysis (PCA) conducted with the log-transformed aligned reads per gene. (**C**,**D**) Venn diagram (**C**) and MA-plots (**D**) showing differential gene expression from the three major inter-group comparisons (HCC-T vs. HCC-NT; HCC-T vs. HL; HCC-NT vs. HL). For MA-plot construction, a gene was considered to be differentially expressed between groups at an absolute log_2_ FC > 2 or <−2 and a FDR of 1% (moderated t-test; Benjamini-Hochberg procedure).

High-throughput cancer studies usually compare tumour tissue to matched non-tumour samples from the same patients. To evaluate if inclusion of an additional reference group consisting of healthy liver tissue samples provides the opportunity to increase the statistical resolution between tumour and adjacent non-tumour tissue, we included 34 healthy liver (HL) RNA-Seq data sets from The Genotype-Tissue Expression (GTEx) project^23^ in our analysis (Fig. 1A). Combined evaluation of all three groups using principal component analysis (PCA) revealed the partial overlap between tumorous HCC (HCC-T) and adjacent non-tumour (HCC-NT) transcriptomes and demonstrated that HL samples formed a separate cluster (Fig. 1B).

Mediating the concern of a potential batch effect when comparing GTEx and TCGA data, the distribution of aligned reads over all genes appeared very similar in the TCGA and the GTEX data (Supplementary Figure S1). Since our main focus was on the expression of immune cell type-specific marker genes, we restricted the expression data to these genes and performed additional PCA. We did not observe a partitioning of the three sample classes (HCC-NT, HCC-T, HL) on the level of the principal component PC1 that explained the largest amount of variation (23.5%; Supplementary Figure S2). In contrast, the variance components that explained a smaller amount of variation (PC2-5; 10–3.4%) led to a distinct clustering of the samples. These findings exclude the presence of a major batch effect interfering with our analysis.

Pearson’s correlation analysis furthermore indicated the highest transcriptional divergence (*R* = 0.91) between HL and HCC-T, whereas HL and HCC-NT transcriptomes had a smaller correlation-based distance (*R* = 0.93) and HCC-T and HCC-NT were most similar (*R* = 0.97) (Supplementary Figure S3). Comparison of common and differentially expressed genes documented that the majority of transcripts were commonly expressed in all three groups (11,899) and identified 1,889 uniquely expressed transcripts for HCC-T, 6 for HCC-NT and 38 for HL samples (Fig. 1C and Supplementary Table 2). As shown in Fig. 1D, statistically significant transcriptome changes (FDR < 0.01; log_2_ FC > 2 and <−2) were more abundant between HCC-T and HL (2,648 transcripts) followed by HCC-NT and HL (1,874 transcripts) and HCC-T and HCC-NT (1,209 transcripts). These results document that patient-derived HCC-T and HCC-NT transcriptomes were much closer related to each other than patient and HL transcriptomes. To exclude that the proximity between HCC-T and HCC-NT transcriptomes negatively affects the overall resolution and to reveal shifts in immune cells and immune activities with regard to healthy liver tissues, we chose to utilize all three data sets for our analysis.

**Figure 2:**
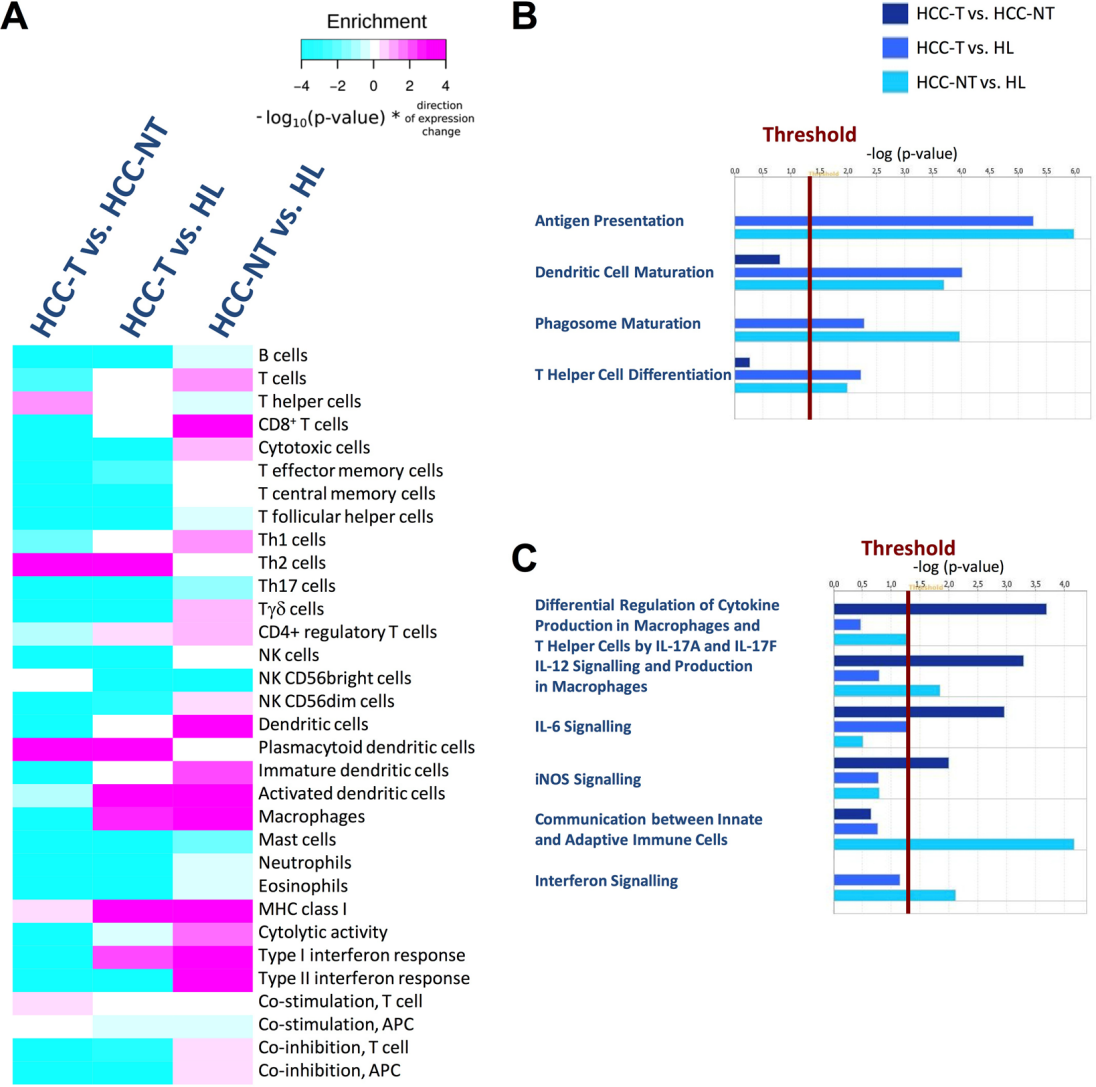
Healthy livers are a critical reference group for identifying differentially regulated immune cell types and pathways in HCC. (**A**) A gene set enrichment analysis was performed to detect coordinated changes of gene expression in gene sets of immune cell type-specific marker genes. Three inter-group comparisons were performed (HCC-T vs. HCC-NT; HCC-T vs. HL; HCC-NT vs. HL). Colour intensity reflects the log_10_ transformed p-value from the enrichment analysis. Magenta colour indicates higher expression compared to the reference group while cyan colour indicates weaker expression compared to the reference group. (**B**,**C**) Selected immune pathways from an IPA^®^ on the three major inter-group comparisons (**B**) activated pathways shared by HCC-T and HCC-NT; (**C**) pathways activated in either HCC-T or HCC-NT).

To gain insight into different immune cell subpopulations infiltrating HCCs and surrounding non-tumour tissues and to study immune effector activity within these two sites, we resorted to recently published human immune cell-specific gene sets^11,13^. Based on these gene sets and using RNA-Seq data from HCC-T, HCC-NT and HL, we devised an immune cell-type marker enrichment approach. Specifically, we calculated the infiltration of 24 different immune cell types and determined the activity of eight mechanistic immune functions that are linked to efficient anti-tumour immune responses (antigen presentation, cytolytic activity, interferon signalling and co-stimulation or co-inhibition of T and antigen presenting cells (APCs)). Consistent with the fact that liver cancer usually arises in a hyper-inflamed tissue context and that immune surveillance is often reduced in solid tumours, we found that most immune cell types such as B and T lymphocytes, NK (natural killer) cells, APCs, mast cells and granulocytes were more abundant in the surrounding liver tissue than in HCCs (Fig. 2A, HCC-T vs. HCC-NT). However, HCCs had more T helper, Th2 and plasmacytoid dendritic cells than the surrounding tissue. With respect to immune activity, marker enrichment analysis revealed that HCCs had more major histocompatibility complex class I (MHC I) gene expression and increased T cell co-stimulation but less cytolytic activity, less co-inhibition of T cells and APCs and less type I as well as type II interferon response. Interestingly, comparison between HCC-T vs. HL samples established that most immune cell types either were reduced in or not changed between HCCs and healthy livers. However, HCCs contained more Th2 cells, regulatory CD4^+^ T cells, activated and plasmacytoid dendritic cells, and macrophages than normal livers. Finally, we calculated differences in the immune contexture between HCC-NT and HL samples. This analysis documented that in tissues surrounding HCC, immune cell subsets required for an effective cytotoxic anti-cancer response such as T cells, CD8^+^ T cells, cytotoxic cells (representing CD8^+^ T cells, Tγδ and NK cells), Th1 cells, Tγδ cells and APCs were highly increased (Fig. 2A, HCC-NT vs. HL). Likewise and confirming the Th1-like immune activation pattern in tumour-adjacent patient samples, MHC I gene expression, cytolytic activity and type I and II interferon response all were strongly augmented (Fig. 2A, HCC-NT vs. HL).

These findings demonstrate a well-defined immune cell compartmentalization between HCCs and surrounding non-tumour tissues. While T helper and in particular, Th2 cells increased in HCCs, immune cells critical for an effective anti-cancer response, such as Th1-specific T cells, cytotoxic cells and dendritic APCs, were reduced. Our analysis thus delineates a very clear picture of the immune contexture of HCC and reveals that HCC is characterised by Th2-skewed tumours with low cytolytic and antigen-presenting activity, which are in turn surrounded by Th1-type hyper-inflamed liver tissue.

The determination of global transcriptome changes between HCC-T, HCC-NT and HL also provides the opportunity to identify specific pathways that are deregulated in each tissue set. To gain more insight into the deregulation of immune response-related programs, transcripts from all three groups were subjected to Ingenuity Pathway Analysis (IPA^®^). IPA^®^ validation unveiled that inclusion of HL samples facilitated the discovery of pathways that were deregulated in both HCCs and surrounding liver tissues. For example, when comparing HCC-T vs. HCC-NT samples, the deregulation of pathways responsible for antigen presentation, dendritic cell maturation, phagosome maturation and T helper cell differentiation was not detected. By contrast, when we applied IPA^®^ to HCC-T vs. HL or HCC-NT vs. HL RNA-Seq data, all four pathways had high activation scores (Fig. 2B and Supplementary Figure S4). This indicates that the gene activity of major constituents of these pathways is significantly increased in both HCC and surrounding non-tumour tissues. Applying IPA^®^ to HCC-T and HCC-NT samples also revealed several immune regulatory differences between these two sites. For example, HCC-NT exhibited strong IL-17A/IL17F-dependent activation of macrophage and T helper cell cytokine production and an increase in IL-6 signalling in comparison to HCC-T (Fig. 2C and Supplementary Figure S5). Directly in line with the increased infiltration of different cytolytic and antigen presenting cells in tissues surrounding HCC, IPA^®^ detected higher activity of pathways responsible for the communication between innate and adaptive cells in HCC-NT samples that was not apparent in HCC-T or HL. Also directly confirming previous marker enrichment results, IPA^®^ recorded the up-regulation of interferon signalling in tumour surrounding tissues in comparison to healthy livers (Fig. 2C and Supplementary Figure S6). We thus conclude that pathways regulating antigen presentation, dendritic cell maturation, phagosome maturation and T helper cell differentiation are upregulated in both HCCs and surrounding non-tumour tissues.

### Analysis of the immune contexture in different HCC risk factor groups and tumour stages

**Figure 3:**
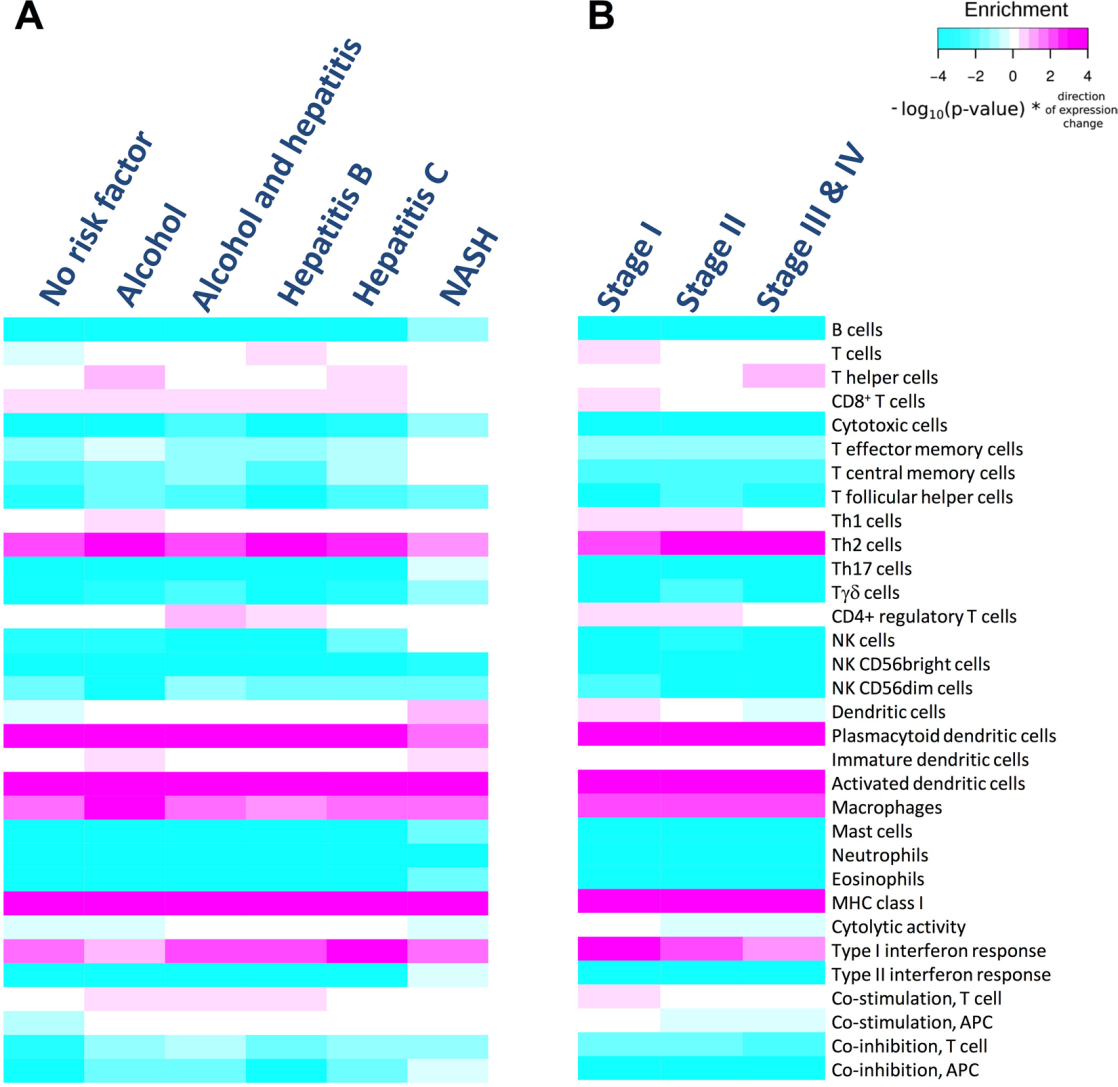
HCC etiology and tumour stage are not linked to major changes in the immune contexture. (**A**,**B**) Immune cell type marker enrichment analysis to compare gene expression between HCC samples grouped for etiology (**A**) and tumour stage (**B**). HL was used as the reference group.

To investigate the immune TME in different HCC etiologies, we divided 371 TCGA patient samples into six groups consisting of patients with no risk factor, alcohol abuse, hepatitis B, hepatitis C, hepatitis and alcohol abuse and non-alcoholic steatohepatitis (NASH) (Supplementary Table 3). Using healthy liver samples as a reference group, we next applied the immune cell type marker enrichment analysis. As shown in Fig. 3A, B lymphocytes, cytotoxic cells, T follicular helper, Th 17 and Tγδ cells as well as NK cell subsets, mast cells, neutrophil and eosinophil granulocytes and also type II interferon response and co-inhibition of T and APC cells were decreased across all HCC etiologies when compared to healthy liver tissues. In contrast, all HCC samples contained more Th2 cells, plasmacytoid and activated dendritic cells as well as macrophages and exhibited increased MHC I gene expression and type I interferon response than normal livers. With regard to all other classifiers and with the exception of a few deviations in the alcohol abuse and NASH groups, no or only subtle differences were recorded across all six HCC etiologies. The lack of major changes in immune cell infiltration and immune activity patterns across HCCs of different etiology is likely to reflect a shared general characteristic of HCC to adapt a TME containing reduced numbers of immune cells and exhibiting less cytolytic, Type II interferon and co-stimulatory/-inhibitory pathway activity patterns.

To next determine whether differences in immune cell infiltration are associated with different HCC disease stages, we calculated the enrichment of different immune cell subsets using samples from early (stage I), intermediate (stage II) and late stage (stage III and IV) HCC patients. While 18 immune cell types showed no good correlation with the analysed tumour stages, the marker enrichment analysis revealed reduced numbers of T cells, CD8^+^, Th1 as well as CD4^+^ regulatory T cells and dendritic cells in samples from more advanced HCC patients (Fig. 3B). With regard to immune activity patterns, late stage tumours exhibited less cytolytic activity, less type I interferon responses and reduced co-stimulation of T cells and APCs. Conversely, late stage tumours contained more T helper and Th2 cells. We conclude that HCC progression entails a gradual loss of anti-cancer immune surveillance potential with late stage HCCs having a T helper cell-dominated and a Th1 anergic TME and a reduced ability to support Th1, cytolytic and Type I interferon signalling activities.

### The immune contexture of HCC is associated with patient survival

**Figure 4:**
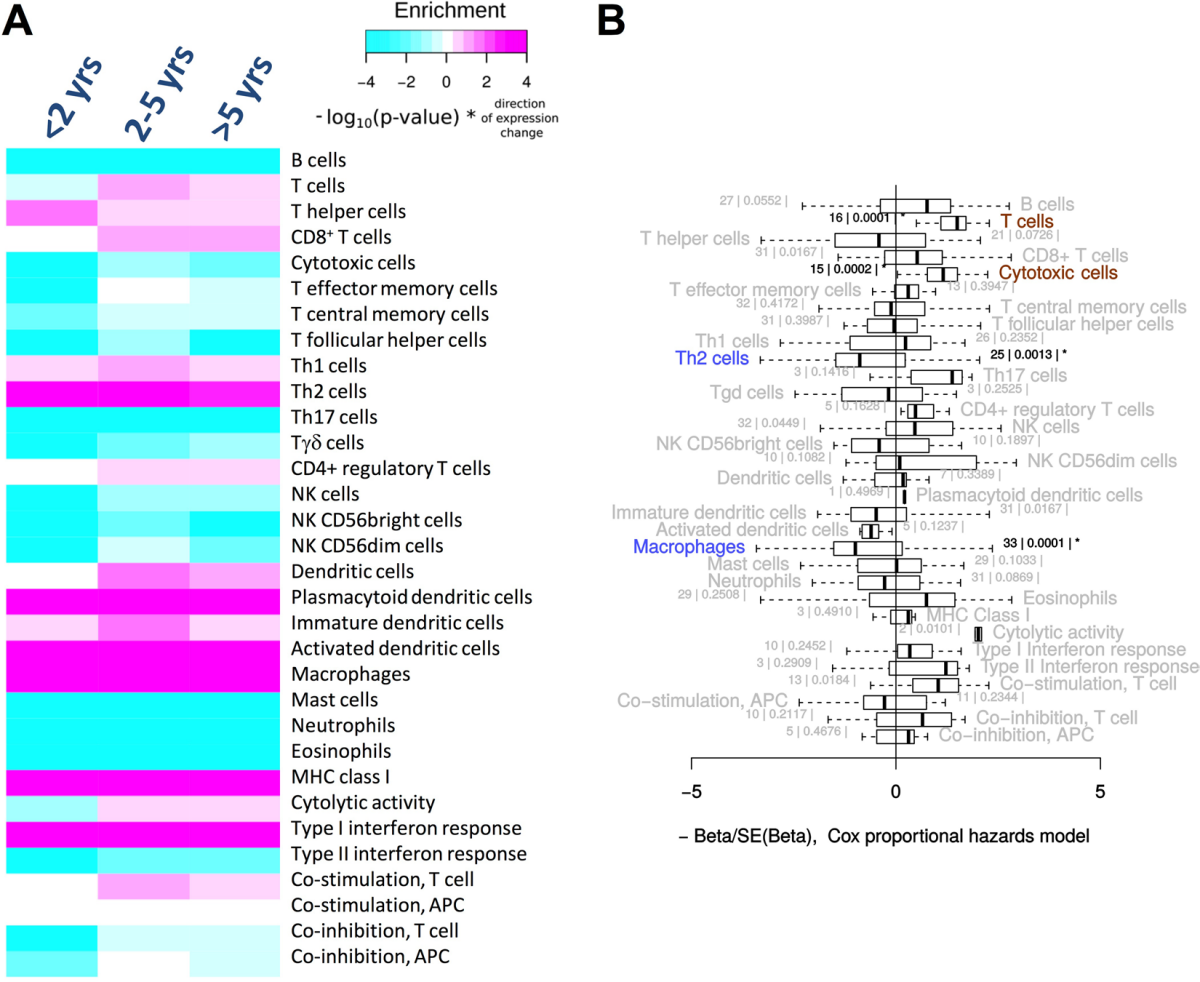
Survival of HCC patients depends on the immune contexture. (**A**) Immune cell type marker enrichment analysis to compare gene expression between HCC samples grouped for survival. Increased expression of marker genes for T cells, CD8^+^ T cells, CD4^+^ regulatory T cells, dendritic cells, cytolytic activity and T cell co-stimulation was observed in patients with favourable survival. HL was used as the reference group. (**B**) Association of immune cell type marker gene expression with survival. For each immune cell marker gene, association with survival in the TCGA data was estimated by Cox proportional hazards models. The distribution of z-scores per cell type category is shown in a boxplot. For each cell type category, consistent association of marker gene expression with survival was assessed. Marker gene sets for T cells, cytotoxic cells, Th2 cells and macrophages were significantly associated with survival at a family-wise error rate (FWER) of 5% indicated by “*” and coloured characters. These gene sets were combined to build a prognostic score (Fig. 5). Blue = higher expression corresponds with shorter survival, brown = higher expression corresponds with longer survival. The number of genes within a gene set and the p-value from the test of consistent association with survival (in vertical bars) are indicated for each cell type.

The infiltration of solid tumours with T cells, APCs and innate immune cells and the presence of immune activities have been directly linked to patient survival in various cancers^11–13^. To investigate the association between the infiltration by different immune cells and clinical outcome, we compared the tumour immune contextures of samples from patients with good (survival > 5 years,), intermediate (survival between 2 and 5 years) and poor outcome (survival < 2 years). To avoid outliers produced by direct post-surgery effects, data sets of patients who died within 30 days after surgery were excluded. Notably, we observed considerable differences across the three groups and particularly between patients with favourable and poor survival (Fig. 4A). Generally, infiltration by immune effector cells such as T cells, CD8^+^ T cells, Tγδ cells and NK cells was increased in samples from patients with good survival relative to those with poor survival.

In order to identify statistically significant associations with survival, we performed a Cox regression on the expression of immune cell type marker gene sets and survival data (Fig. 4B). A variety of immune cells including T cells, CD8^+^ as well as cytotoxic cells and NK cells were positively, while T helper/Th2 cells, immature dendritic cells and macrophages were negatively associated with survival (p < 0.05). After adjustment for multiple testing, T cells, cytotoxic cells, Th2 cells and macrophages were associated at a family-wise error rate (FWER) of 5%. Likewise and although MHC class I expression was not associated with survival, immune defence activity patterns such as cytolytic activity and co-stimulation of T cells were positively associated with survival. From this analysis, we conclude that HCCs bearing increased numbers of adaptive and innate immune cell subsets and supporting a Th-1-type immune response have a favourable survival. Conversely, intra-tumour invasion of Th2 cells and macrophages are inversely associated with patient survival. Of note is also that a similar pattern was obtained when an unrelated HCC patient cohort^24^ was analysed in the same way (Supplementary Figure S7).

### A prognostic immune signature for HCC

**Figure 5:**
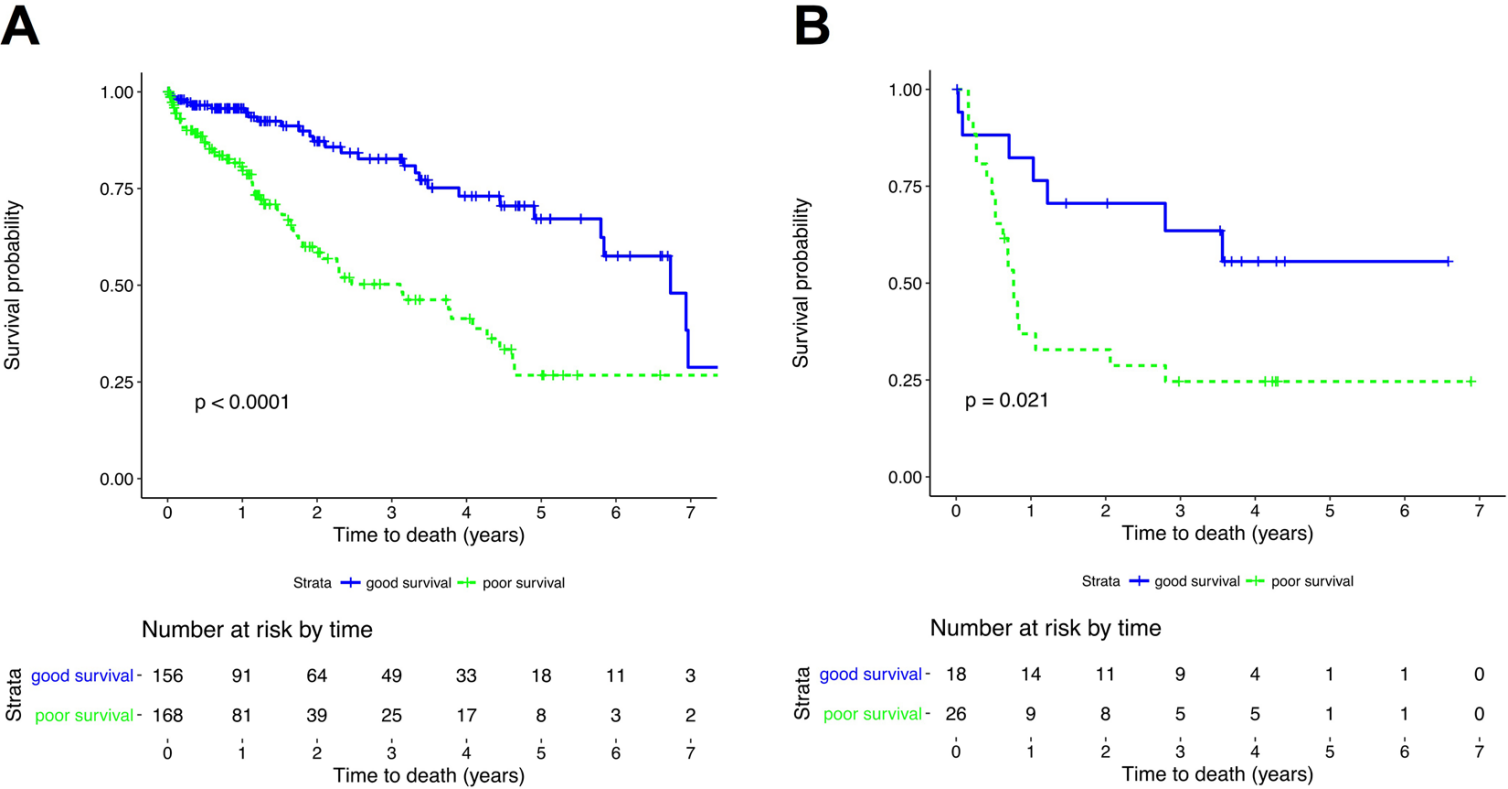
An immune gene signature comprising marker genes for T cells, cytotoxic cells, Th2 cells and macrophages predicts survival in HCC patients. (**A**) Kaplan-Meier analysis showing the differential survival of the TCGA HCC patient cohort according to the novel immune gene-based prognostic score. The prognostic score was defined using previously identified immune cell type gene sets (T cells, cytotoxic cells, Th2 cells and macrophages). Groups of patients with predicted good and poor survival were built based on the gene-wise statistics of the proportional hazards model (Fig. 4B) using a scoring system. Kaplan-Meier estimators were calculated for both groups. Significance in differential survival between both groups was determined using a log-rank test. (**B**) Validation of the gene signature in an independent set of HCC samples confirmed that this score distinguishes between patients with good and poor survival. All analyses were conducted as in “A”.

Having demonstrated that the HCC immune contexture is linked to clinical outcome, we sought to establish a prognostic immune gene signature that distinguishes between groups with distinctly different survival. To this end, we combined the previously established four significant gene sets from the Cox regression analysis to a common immune gene signature (T cells, cytotoxic cells, Th2 cells and macrophages, genes listed in Supplementary Table 4). Application of this 89 immune gene signature divided the TCGA HCC cohort into patients with poor and favourable survival (Fig. 5A; hazard ratio [HR] for “poor”: 3.033; 95% confidence interval [CI], 1.918 to 4.797; p < 0.0001). Directly confirming the predictive power of the established immune gene signature, analysis of an unrelated HCC patient cohort^24^ also accurately predicted patient survival (Fig. 5B; hazard ratio [HR] for “poor”: 2.704; 95% confidence interval [CI], 1.126 to 6.495; p = 0.021). In addition, when we restricted the signature to gene sets specific for T cells, cytotoxic cells and macrophages, this score performed even better (64 genes; Supplementary Figure S8, genes listed in Supplementary Table 4).

In order to compare our gene signature with the hitherto state of the art, we tested three previously published gene sets: First, we applied the CIBERSORT algorithm^25^ to our TCGA patient dataset. Application of CIBERSORT to TCGA HCC samples (which was not done in the original pan-human cancer study by Gentles *et al*.^12^) and using a resampling test (for details see^25^), CIBERSORT reported that only 81 of 405 tested HCC and liver samples contained any type of immune cells (Supplementary Figure S9). Since it is not likely and plausible that 80% of the TCGA samples do not contain any immune cells, we did not consider the CIBERSORT method as a valuable approach for analysing HCC samples. Second, we applied two prognostic immune signatures previously published by Chew *et al*.^16,26^ to the TCGA data set. Both signatures reached a lower significance level (Supplementary Figure S10; hazard ratio [HR] for “poor”: 1.795; 95% confidence interval [CI], 1.149 to 2.803; p = 0.0092^16^ and hazard ratio [HR] for “poor”: 1.83; 95% confidence interval [CI], 1.175 to 2.848; p = 0.0067^26^) in comparison to our immune signature indicating a stronger association of our gene set with survival (Fig. 5A). Finally, we tested an immune classifier set of 108 genes used to classify HCC tumour immune milieus that was published during the revision of our paper^27^. This signature achieved similar significance levels to predict survival (Supplementary Figure S10; hazard ratio [HR] for “poor”: 2.71; 95% confidence interval [CI], 1.706 to 4.304; p < 0.0001).

These results clearly demonstrate that the 89 and the 64 immune gene signatures have general utility for prognostic HCC stratification suggesting that RNA-Seq data analysis of patient biopsies with these signatures can be directly used to guide future targeted and/or immune therapies.

## Discussion

Next generation bioinformatics analysis of high-dimensional data sets is increasingly used for the comprehensive and unbiased characterisation of cancer TMEs and provides the opportunity to precisely characterise the infiltration of different immune cell types and immune activity patterns within tumours^11–13^. Here we report the first comprehensive bioinformatics analysis of the immune TME for human HCC. Our results demonstrate that different HCC etiologies and stages are not associated with major changes in the immune contexture and reveal that the nature and composition of tumour-infiltrating immune cells correlate with patient survival.

Strikingly and although next generation bioinformatics have been extensively used for characterising the immune TME of most major human malignancies^11–13^, no comprehensive immunome analysis for HCC has been published. Equally, it has been completely unknown if different HCC etiologies or disease stages have different immune contextures. And most importantly, it has still remained to be established whether the immune contexture of HCC correlates with patient survival. Interestingly, the excellent bioinformatics analysis by Gentles and colleagues^12^, which covers all major human cancer subtypes, did not include human HCC, and the CIBERSORT tool that was developed in this study to predict patient survival did not produce plausible results when we used this algorithm on human TCGA HCC data. To close this gap and to provide detailed insight into the immune TME of human HCC, we established a straightforward bioinformatics approach and applied this methodology to human HCC integrating data from tumour samples, surrounding non-cancerous tissues and healthy livers.

Historically, most cancer studies compared tumour tissue to matched non-tumour samples from the same patient. By comparing patient-derived (HCC-T and HCC-NT) with healthy liver (HL) samples, we found that the transcriptomes of tumour and surrounding tissue samples formed partially overlapping clusters having close transcriptional proximity, while healthy liver probes clustered separately and were transcriptionally less related to HCC-T and HCC-NT patient samples. Regarding potential and unwanted batch effects arising during the comparison of different sample groups (HCC-T, HCC-NT and HL), both analysis of the overall distribution of the number of aligned reads per gene between the three groups and a PCA on the level of immune cell marker gene expression demonstrated the absence of major batch effects.

Highlighting that the inclusion of non-patient healthy liver tissue samples as a reference group provided additional advantages, we were able to uncover common immune-related switches shared between HCC-T and HCC-NT that would not have been identified by comparing tumours with adjacent non-tumour tissues. The partial overlap and close proximity of patient-derived HCC-T and HCC-NT samples and the opportunity to reveal mRNA expression patterns that are common to patient-derived probes but absent from healthy tissues highlights that the analysis of high-dimensional sequencing data greatly benefits from the inclusion of healthy tissue samples.

The first important feature emerging from our analysis is that HCC liver tissues are immunologically compartmentalized into immune compromised tumour areas that are surrounded by hyper-inflamed non-tumour tissues. Comparison of patient-derived and healthy samples clearly demonstrated that most immune cell subsets required for an efficient anti-tumour immune response were decreased in tumour samples when compared to tumour surrounding areas and also with regard to healthy livers. By contrast, gene signatures defining T helper and Th2 cells were strongly increased in HCCs. Together with the low cytolytic and type I and type II interferon signalling activity recorded in HCC samples, these results reveal that HCC consists of a highly compromised, Th2-skewed immune milieu that lacks sufficient Th1-specific and cytolytic immune properties. Because targeted therapies and in particular immune therapies can be assumed (at least to some extent) to depend on the composition of the immune TME, additional research is required to investigate if such strategies can indeed modulate the immune contexture of HCC and thus enhance the potential of cancer therapies.

Previous studies on the global transcriptome of HCC have proposed molecular classifications based on gene expression profiling and exhibited correlation between distinct molecular subclasses and clinical parameters^28,29^. However, while clinical TNM-based and molecular features have been traditionally used to stratify different tumour subtypes, stages and clinical outcome, emerging evidence suggests that the immune contexture might be very useful or even superior for classifying tumour types and stages and for predicting patient survival^9,11–14,30^. Although we found no major changes in the immune TME of HCCs with different etiologies, our data strongly support the view that the HCC immunome is an excellent classifier for predicting patient survival. Indeed gene set enrichment analysis of HCC patient data established that the immune contexture of HCC did not or only very moderately differ among HCC etiologies. However, when we compared the immune contexture of different HCC tumour stages, we observed the selective reduction of (CD8^+^) T cells and dendritic cells, which was accompanied by an increase in T helper cells and a decrease in cytolytic and co-stimulatory activity in samples from more advanced patients. This clearly indicates that HCC gradually loses the ability to mount an effective Th1 and cytolytic immune response upon disease progression. Of note is also that the expression profiles for 18 immune cell types remained unchanged across different tumour stages. The lack of major alterations in 18 immune cell subsets between early and late HCCs suggests that these cells are not relevant for tumour progression. However, since the here applied immune cell-specific gene sets were limited to 24 major immune cell types, our analysis may not have recognized differences in more specialized immune cell subtypes such as differently polarized macrophages or myeloid-derived suppressor cells, that are known to be mechanistically linked to HCC tumour progression and stage^31–33^. In the future, gene sets for these cell types have to be defined yielding additional insights about the correlation of such subsets with HCC tumour stage and progression.

Another question our study cannot address due to lacking clinical information is whether pre-treatment of HCC patients such as hepatic resection or transarterial chemoembolization affects the composition of the immune contexture. This remains to be uncovered and will likely impact future clinical trials investigating either different treatment sequences or combinations of locoregional approaches and immunotherapies. In this regard, the STORM trial, which had surprisingly not found a benefit for an adjuvant treatment with sorafenib after resection or ablation in HCC patients^34^, serves as a historical example of a study on the dependency between locoregional and adjuvant systemic treatment.

Our study also established a clear association between overall survival, the invasion of specific immune cells and the strength of immune activity patterns in HCC. Overall, we noticed a positive association of an immunostimulatory TME with survival and vice versa. In this respect, we identified gene sets for T cells, CD8^+^ as well as cytotoxic cells, Th2 cells, NK cells, immature dendritic cells, macrophages, cytolytic activity and T cell co-stimulation that were associated with survival. Our findings thus extend and are directly supported by previous studies reporting superior survival rates in HCC patients with an inflammatory TME^16^ and vice versa in case of an immunosuppressive microenvironment^35^. In particular, tumour infiltration by T and NK cells^26^ as well as tumour-associated antigen-specific CD8^+^ T cell responses^36^ have been reported to correlate with survival in HCC. Our analysis thus provides clear additional evidence that the infiltration by Th1-type immune cells and the presence of a cytolytic TME are directly linked to a favourable clinical outcome.

Based on our findings, we introduce a novel prognostic scoring algorithm comprising immunological markers for T cells, cytotoxic cells, Th2 cells and macrophages. When validated using an independent HCC data set, this signature stratified patients according to survival suggesting the general utility of this signature for future applications. More importantly, our gene signature was more significantly associated with survival than the two signatures published by Chew *et al*.^16,26^. The immune gene set simultaneously proposed by Llovet and colleagues^27^, which shares only 8 genes with the here established signature, achieved similarly high significance levels highlighting the appropriateness of using immune-related gene signatures to stratify patients.

Taken together, our study emphasizes the relevance of the immune TME for the outcome of human HCC and provides the first comprehensive report about the relevance of different immune cell subsets and immune activity patterns for this disease. The here presented bioinformatics approach also represents a straightforward methodology for analysing other human malignancies and will be highly useful for monitoring and guiding future clinical studies.

## Methods

All statistical analyses were conducted within the statistical programming environment R (v3.3)^37^. The R-code used for the immune cell type marker enrichment analysis, the Cox regression, and the prognostic score is available through GitHub (https://github.com/ssehztirom/foerster-hess-immune-contexture-2018).

### Gene expression data

Gene expression measured by mRNA-Seq in HCC from 371 patients is available in The Cancer Genome Atlas (TCGA). For 50 of these patients, gene expression measured in adjacent liver tissue is available (RNASeqV2; 2015/12/17). Gene expression data measured by mRNA-Seq in tumour-free post-mortem liver samples of 34 donors is available in the Genotype-Tissue Expression project (GTEx; v.4). Data was downloaded from the TCGA data portal (https://tcga-data.nci.nih.gov/tcga/; “…rsem.genes.results”) and from the GTEx portal (http://www.gtexportal.org/home/; “…gene_reads”). Venn diagrams were built for each group considering transcripts as being expressed when aligned gene reads were recorded in ≥10 out of 30 RNA-Seq libraries.

### Merging of TCGA with GTEx data

To harmonize TCGA and GTEx data sets, GENCODE annotations (v.18) used for the quantification of the GTEx data were assigned to the corresponding UCSC gene annotations using the genome annotation file (GTF) provided by the GTEx project (gencode.v18.genes.patched_contigs.gtf). Merging GTEx and TCGA data resulted in 16,776 common gene annotations shared between both data sets (Supplementary Table 5).

### Clinical data and subgroup definition

For each sample, donor characteristics were extracted from TCGA (biospecimen_sample_lihc.txt, clinical_patient_lihc.txt) as well as GTEx data sheets (GTEx_Data_2014-01-17_Annotations_SampleAttributesDS.txt) and are listed in Supplementary Table S3. Since age was indicated in decades in the GTEx data set, patient age from the TCGA data was also stratified using 10 year periods as age classifiers. For statistical analysis, patients were grouped according to survival (<2, between 2 and 5 and >5 years; patients with a survival <30 days were excluded), known risk factors (alcohol abuse, hepatitis B, hepatitis C, hepatitis B and/or C and alcohol abuse, non-alcoholic steatohepatitis (NASH) and no risk factor (i.e patients for who the etiology was not clearly defined)) and TNM/UICC tumour stage (stage I, stage II and stage III & IV). Patients with incomplete medical information were excluded.

### Detection of differential expression

Differential mRNA expression was inferred using linear models. For testing differential expression in the risk factor, survival and tumour stage subgroups, TCGA HCC samples were compared to healthy liver samples from GTEx. Age and gender were always included as covariates in addition to the factor of interest. Linear models were fit using limma (v3.28)^38^. After normalizing for sequencing depth, trimmed mean (TMM) normalization^39^ was implemented in EdgeR (v3.14)^40^. The limma routine “voom” was employed to log transform the RNA-Seq data and to estimate precision weights for each gene which were used for improved accuracy of the linear model fitting in limma^41^. Pseudo-replication resulting from tumour samples and samples of adjacent liver tissue stemming from the same individual was addressed by estimating the intra-patient correlation of gene expression using the limma routine “duplicateCorrelation”. Differential expression was assessed using moderated t-tests. Genes that were differentially expressed at a false discovery rate (FDR) < 0.01 and a log_2_-fold change (FC) > 2 and <−2 were considered significant.

### Immune cell type marker enrichment

For identifying different immune cell subsets and immune activity patterns, marker genes were retrieved from previous publications^11,13^ and are listed in Supplementary Table 6. Enrichment of these marker sets was determined using gene set enrichment analysis. To this end, the roast method^42^ implemented in limma^38^ was employed as it allows for enrichment testing between two groups based on a multivariate linear model. The linear models used for the univariate analyses (“Detection of differential expression”) were employed here. In particular, we tested, whether the mean t-statistic in a gene set that is characteristic for a specific immune cell type or a specific activity pattern was significantly shifted from zero in a two-group comparison. Significance was assessed by 10,000 random rotations of the residuals orthogonal to the adjustment variables and by comparing the observed mean t-statistic with the results obtained by the rotated residuals.

### Cox regression to investigate the association of immune cell type marker gene expression with survival

For each marker gene in the immune marker gene list (see “Immune cell type marker enrichment”), association with survival was estimated. Cox proportional hazards models were fitted to the survival data stored in the TCGA using the “coxph” function from the “survival” package (v2.39). The expression of a marker gene was transformed using the voom transformation implemented in limma^41^ and entered the model as covariate. We adjusted for patient age (measured in decades) and gender. After the model fit, z-scores were computed for the gene expression variable. Consistent association with survival within a cell type-specific marker gene set was assessed by comparing the mean of the z-scores within a cell type-specific gene set with an empirical null distribution. The distribution was generated by sampling an equivalent number of z-scores from the total set of z-scores in the total immune cell marker gene set and calculating the mean z-score. Sampling was repeated 10,000 times and p-values indicate the proportion of sampled mean z-scores being more extreme than the observed mean z-scores.

### Ingenuity Pathway Analysis^®^

The networks analyses were conducted with QIAGEN’s Ingenuity Pathway Analysis^®^ (IPA^®^, QIAGEN Redwood City, www.qiagen.com/ingenuity). In all cases p-values and log_2_ FCs of differentially expressed genes (FDR < 0.01 and log_2_ FC > 1 and <–1) were submitted to IPA^®^.

### Building a prognostic score based on immune cell-type marker gene expression

Based on the results of the Cox regression performed with the TCGA data, immune cell groups were selected and the expression of the corresponding marker genes was aggregated using a scoring system. For each cell type marker gene, the individual patient’s score was increased by “1” if the expression was below the median when higher gene expression corresponded with higher risk or if the expression was above the median when higher gene expression corresponded with a lower risk. The prognostic score derived from the TCGA data was validated in an independent data set^24^. Survival probabilities were determined by the Kaplan-Meier method, and comparison between groups was performed by log-rank tests.

## Electronic supplementary material

Supplementary figures

Supplementary tables
