# Supplementary material for: The immune contexture of hepatocellular carcinoma predicts clinical outcome
Source: Sci Rep. 2018 Mar 29;8:5351. doi: 10.1038/s41598-018-21937-2 (PMC5876395; doi:10.1038/s41598-018-21937-2)
Supplement: Supplementary file 1 — Supplementary figures [file 41598_2018_21937_MOESM1_ESM.pdf]

The immune contexture of hepatocellular carcinoma predicts clinical outcome

Friedrich Foerster[1, 2*], Moritz Hess[3*], Aslihan Gerhold-Ay[3], Jens Uwe Marquardt[2], Diana Becker[2], Peter Robert Galle[2], Detlef Schuppan[1, 4], Harald Binder[3], Ernesto Bockamp[1, **]

[1]Institute of Translational Immunology and Research Centre for Immunotherapy, University Medical Centre Mainz, Germany
[2]First Department of Medicine, University Medical Centre Mainz, Germany
[3]Institute of Medical Biostatistics, Epidemiology and Informatics (IMBEI), University Medical Centre Mainz, Germany
[4]Division of Gastroenterology, Beth Israel Deaconess Medical Center, Harvard Medical School, Boston, USA

* These authors contributed equally

** Corresponding author
Ernesto Bockamp, PhD
Address: Obere Zahlbacher Str. 69
Telephone: 0049-(0)6131-179780
Fax number: 0049-(0)6131-177357
Email: bockamp@uni-mainz.de

Supplementary Figures

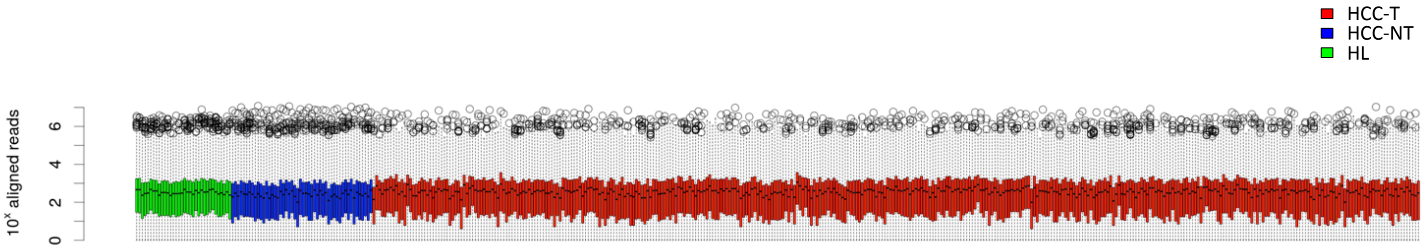

Supplementary Figure S1.

Distribution of the number of aligned reads per gene in 34 tumor-free livers (healthy livers = HL; obtained from GTEx; green), 50 matched samples of tissue adjacent to HCC (HCC-NT; TCGA; blue) and 371 HCCs (HCC-T; TCGA; red). Number of aligned reads were aggregated on the level of UCSC gene annotations and are shown as $\log_{10}$ transformed values.

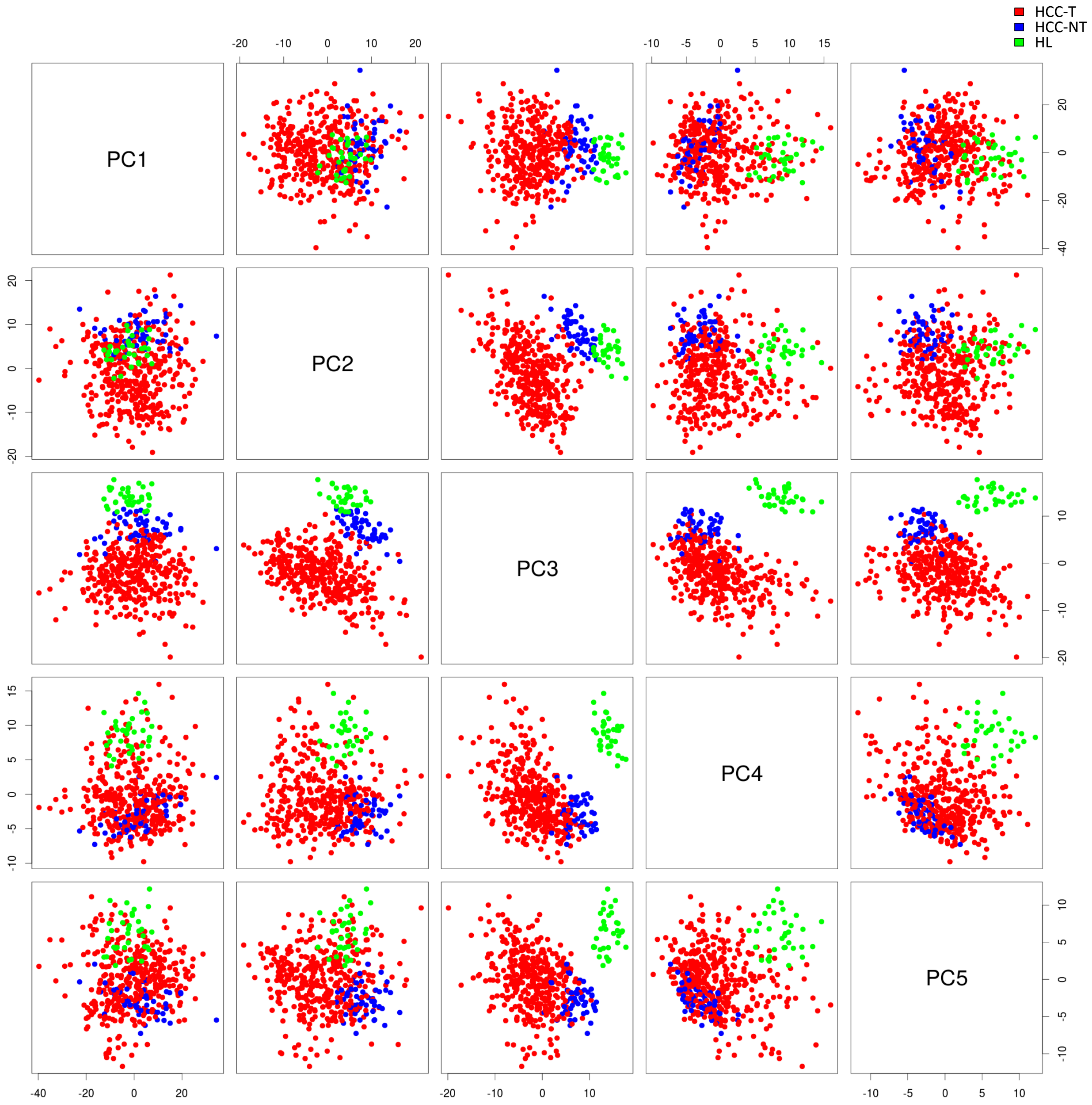

Supplementary Figure S2.

Expression of immune cell marker genes in HL, HCC-NT and HCC-T. Expression is shown on the level of the five variance components (PC1-5) that explain 50% of the expression variation of immune cell type-specific marker genes in healthy liver tissue or HCC. Variance components were obtained by principal component analysis conducted with the $\log_e x$-transformed expression data of 522 immune cell-type specific marker genes in 34 tumor-free livers (healthy livers = HL; obtained from GTEx; green), 50 matched samples of tissue adjacent to HCC (HCC-NT; TCGA; blue) and 371 HCCs (HCC-T; TCGA; red).

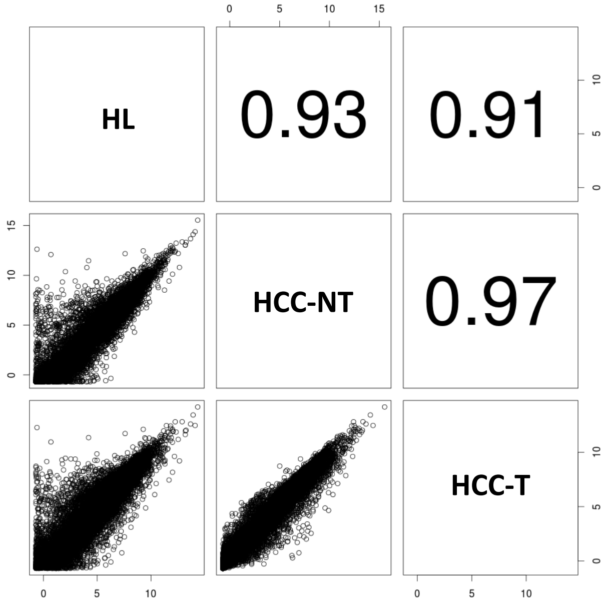

Supplementary Figure S3.
Correlation of global gene expression between HCC-T, HCC-NT and HL samples. Numbers indicate the Pearson correlation coefficient for each comparison.

Antigen Presentation Pathway

HCC-T vs. HCC-NT

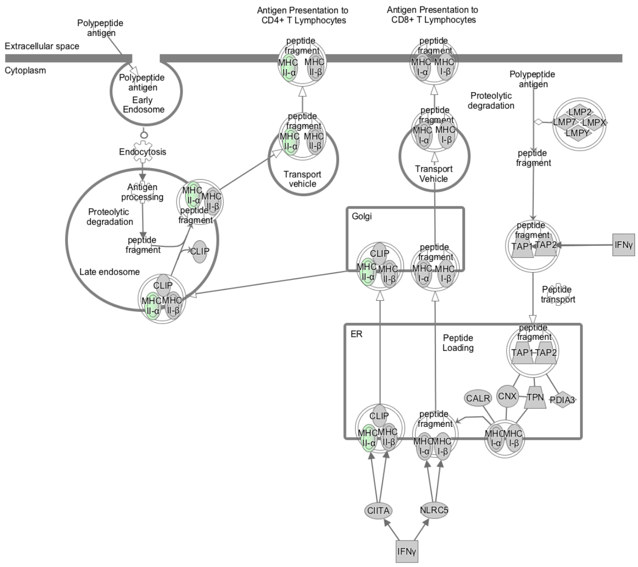

HCC-NT vs. HL

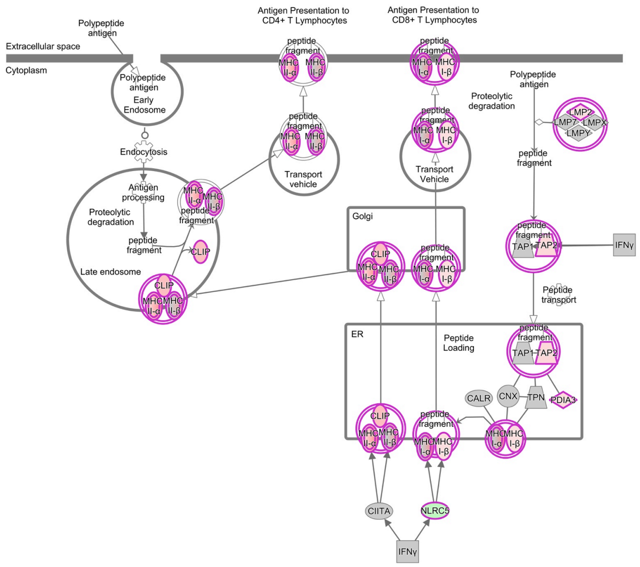

HCC-T vs. HL

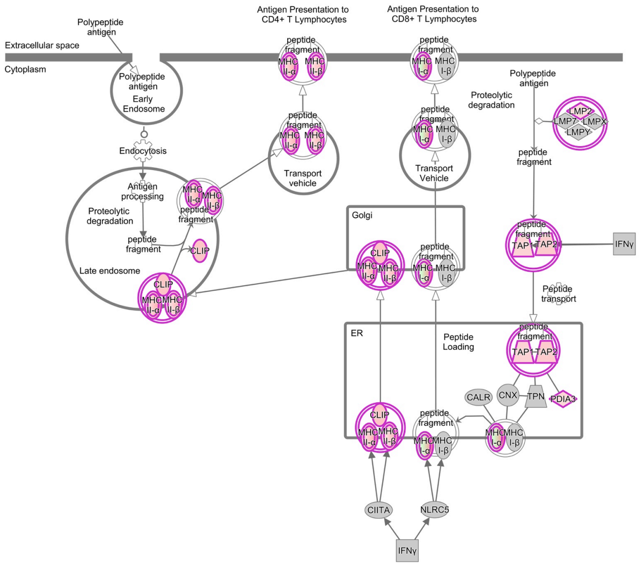

Supplementary Figure S4.
Diagrams of the antigen presentation pathway from an IPA® on differentially expressed genes HCC-T and HCC-NT, HCC-NT and HL as well as HCC-T and HL samples (genes with increased expression in the first group of the two-group comparisons are in red, those of the second group are in green).

Differential Regulation of Cytokine Production in Macrophages and T Helper Cells by IL-17A and IL-17F

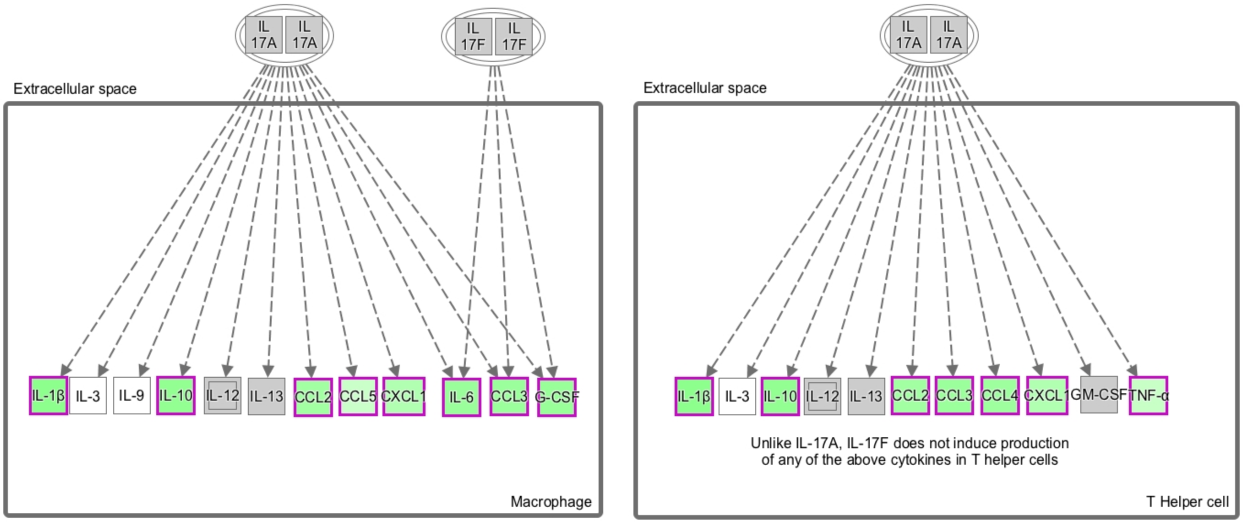

IL-6 Signalling

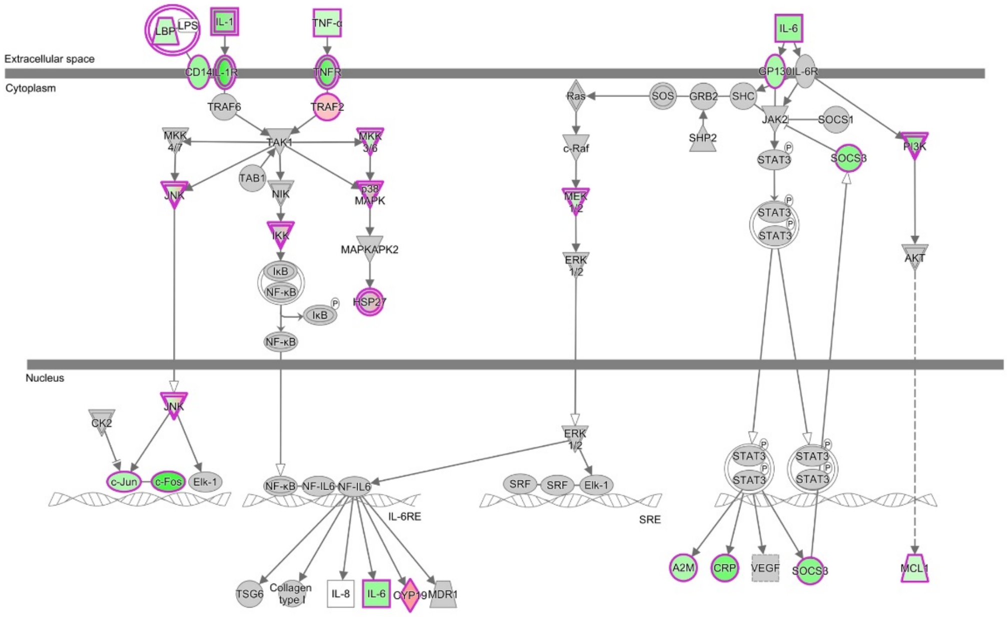

Supplementary Figure S5.
Diagrams of the pathways “IL-17A/IL17F-dependent activation of macrophage and T helper cell cytokine production” and “IL-6 signalling” from an IPA® on differentially expressed genes between HCC-T and HCC-NT samples (genes with increased expression in HCC-T samples are in red, those increased in HCC-NT samples are in green).

Interferon Signalling

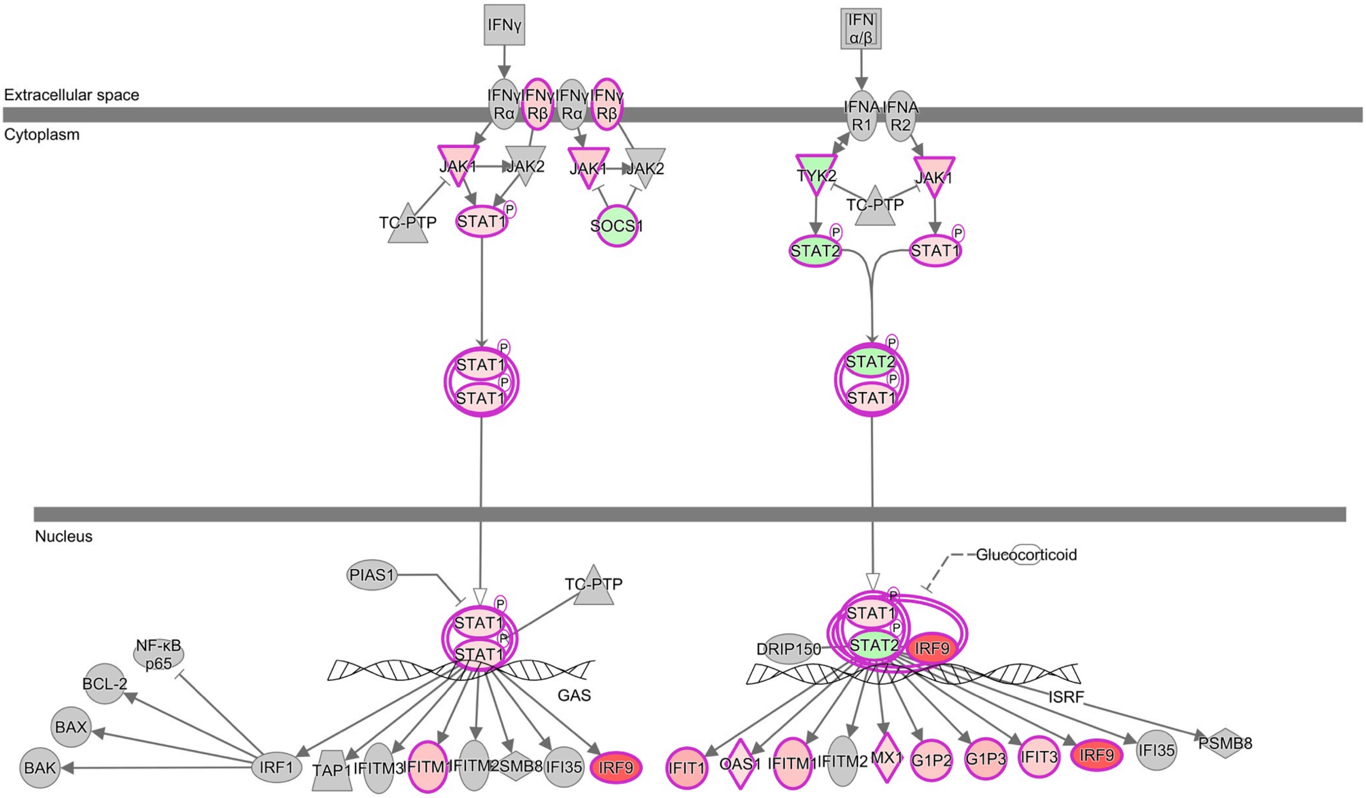

Supplementary Figure S6.
Diagram of the interferon signalling pathway from an IPA® on differentially expressed genes between HCC-NT and HL samples (genes with increased expression in HCC-NT are in red, those increased in HL samples are in green).

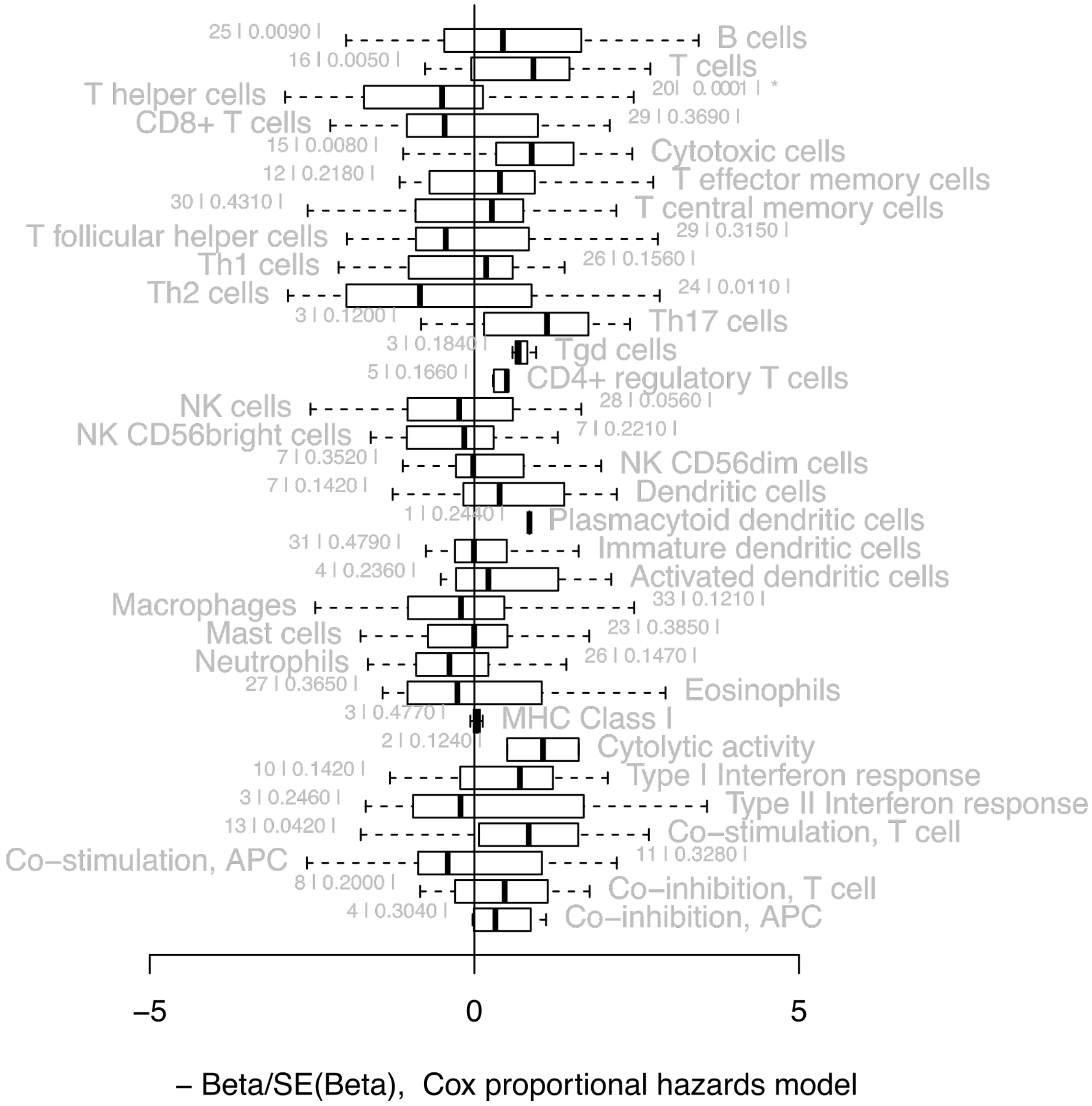

Supplementary Figure S7.
Association of immune cell type-specific marker gene expression with survival in an independent HCC patient cohort. For each immune cell marker gene, association with survival in the TCGA data was estimated by Cox proportional hazards models. The distribution of z-scores per cell type category is shown in a boxplot. For each cell type category, consistent association of marker gene expression with survival was assessed (left of the y-axis = negative association; right of the y-axis = positive association). Association with survival at a family-wise error rate (FWER) of 5% is indicated by "*". The number of genes within a gene set and the p-value from the test of consistent association with survival (in vertical bars) are indicated for each cell type.

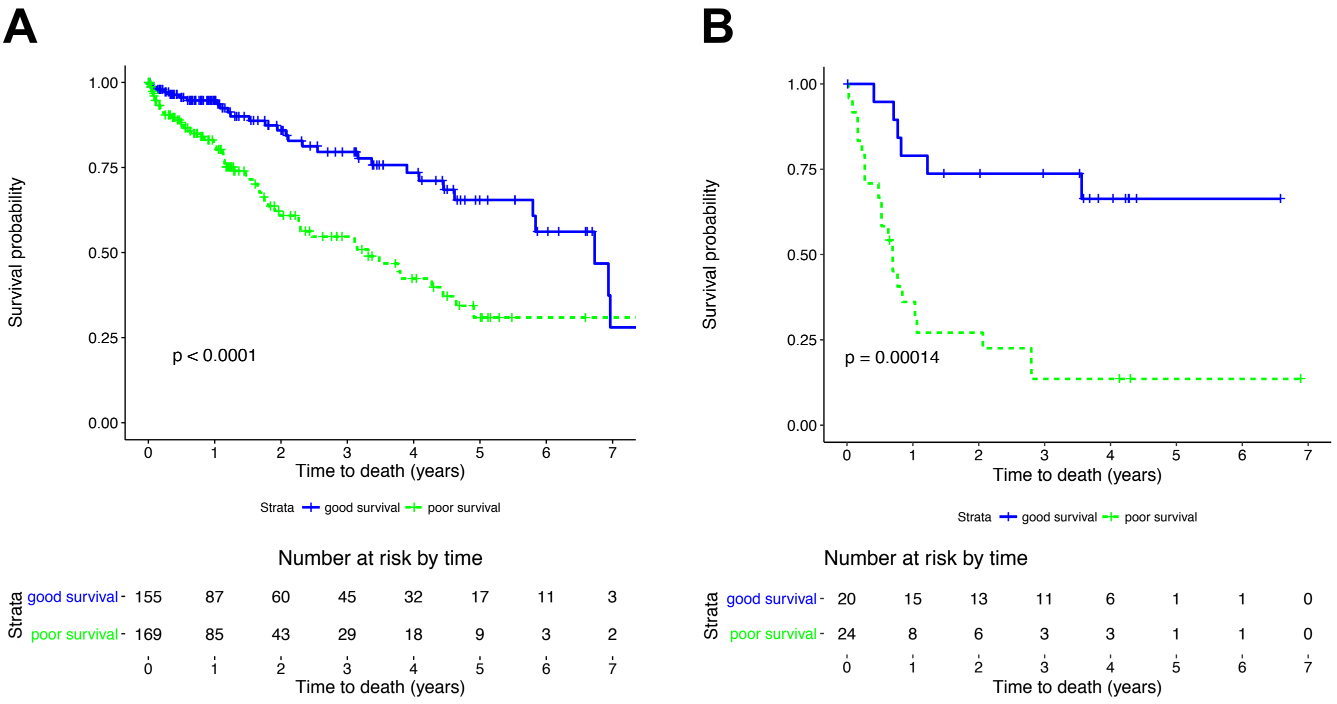

Supplementary Figure S8.

(A) Kaplan-Meier analysis showing the differential survival of the TCGA HCC patient cohort according to the novel immune gene-based prognostic score restricted to the gene sets T cells, cytotoxic cells and macrophages. Kaplan-Meier estimators were calculated for both groups. Significance in differential survival between both groups was determined using a log-rank test.

(B) Validation of the restricted gene signature (T cells, cytotoxic cells and macrophages) in an independent set of HCC samples. All analyses were conducted as in “A”.

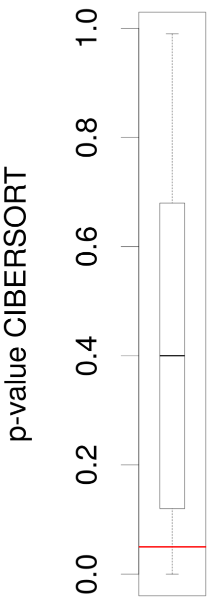

Supplementary Figure S9.
Distribution of p-values returned by CIBERSORT pipeline. The red line indicates a p-value of 0.05, which represents the probability of the null-hypothesis, that there are no immune-cell types contained within the sample.

Chew et al. 2010 | Chew et al. 2012 | Sia et al. 2017

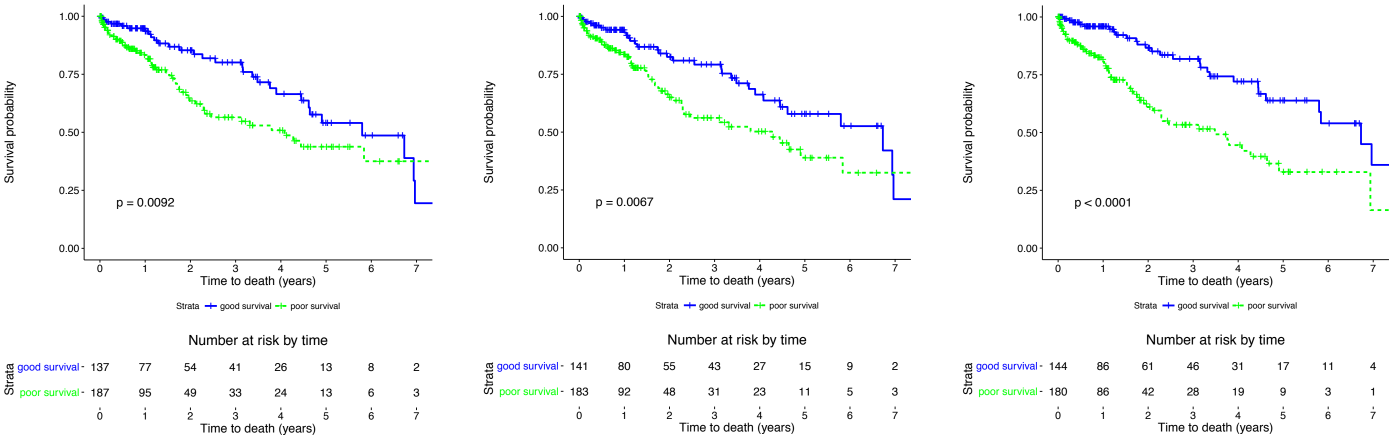

Supplementary Figure S10.

Kaplan-Meier analysis showing the differential survival of the TCGA HCC patient cohort according to three previously published prognostic immune gene signatures (from left to right: Chew et al.[16]; Chew et al.[26]; Sia et al.[27]). Kaplan-Meier estimators were calculated for both groups. Significance in differential survival between both groups was determined using a log-rank test.
